# Wanted

**Published:** 1886-04

**Authors:** 


### WANTED.

A few copies of this JOURNAL for March, for which 25 cents each will be paid cash; or a month's subscription given for each. The demand for the March number was unprecedented, and before we were aware of it the edition was exhausted, execept our office files. The JOURNAL is a booming, louder and louder.

